# Polyphenol Supplementation Did Not Affect Insulin Sensitivity and Fat Deposition During One-Month Overfeeding in Randomized Placebo-Controlled Trials in Men and in Women

**DOI:** 10.3389/fnut.2022.854255

**Published:** 2022-05-09

**Authors:** Bérénice Segrestin, Pauline Delage, Angéline Nemeth, Kevin Seyssel, Emmanuel Disse, Julie-Anne Nazare, Stéphanie Lambert-Porcheron, Laure Meiller, Valerie Sauvinet, Stéphanie Chanon, Chantal Simon, Hélène Ratiney, Olivier Beuf, François Pralong, Naba-al-Huda Yassin, Alexia Boizot, Mélanie Gachet, Kathryn J. Burton-Pimentel, Hubert Vidal, Emmanuelle Meugnier, Nathalie Vionnet, Martine Laville

**Affiliations:** ^1^INSERM, INRAe, CarMeN Laboratory, Claude Bernard Lyon 1 University, Lyon, France; ^2^CRNH-RA, INSERM, INRAe, Hospices Civils de Lyon, Claude Bernard Lyon 1 University, Lyon, France; ^3^Centre Hospitalier Lyon-Sud Service d’Endocrinologie Diabète Nutrition Lyon, Hospices Civils de Lyon, Lyon, France; ^4^CNRS, INSERM, CREATIS, Université de Lyon, INSA-Lyon, Claude Bernard Lyon 1 University, UJM-Saint Etienne, Lyon, France; ^5^Service of Endocrinology, Diabetes and Metabolism, Lausanne University Hospital, Lausanne, Switzerland

**Keywords:** polyphenols, overfeeding, insulin sensitivity, nutritional intervention, insulin resistance, grape polyphenols, gender difference

## Abstract

Two randomized placebo-controlled double-blind paralleled trials (42 men in Lyon, 19 women in Lausanne) were designed to test 2 g/day of a grape polyphenol extract during 31 days of high calorie-high fructose overfeeding. Hyperinsulinemic-euglycemic clamps and test meals with [1,1,1-^13^C_3_]-triolein were performed before and at the end of the intervention. Changes in body composition were assessed by dual-energy X-ray absorptiometry (DEXA). Fat volumes of the abdominal region and liver fat content were determined in men only, using 3D-magnetic resonance imaging (MRI) and magnetic resonance spectroscopy (MRS) at 3T. Adipocyte’s size was measured in subcutaneous fat biopsies. Bodyweight and fat mass increased during overfeeding, in men and in women. While whole body insulin sensitivity did not change, homeostasis model assessment of insulin resistance (HOMA-IR) and the hepatic insulin resistance index (HIR) increased during overfeeding. Liver fat increased in men. However, grape polyphenol supplementation did not modify the metabolic and anthropometric parameters or counteract the changes during overfeeding, neither in men nor in women. Polyphenol intake was associated with a reduction in adipocyte size in women femoral fat. Grape polyphenol supplementation did not counteract the moderated metabolic alterations induced by one month of high calorie-high fructose overfeeding in men and women. The clinical trials are registered under the numbers NCT02145780 and NCT02225457 at ClinicalTrials.gov and available at https://clinicaltrials.gov/ct2/show/NCT02145780 and https://clinicaltrials.gov/ct2/show/NCT02225457.

## Introduction

Unbalanced eating habits with increased consumption of high calorie and fructose rich foods are major contributors to the current obesity epidemics and its metabolic consequences, including type 2 diabetes and metabolic syndrome. Changing lifestyle (reducing energy intake and increasing physical activity) is difficult to maintain in the long term (1) and alternative solutions must be therefore explored to limit the incidence of weight-related metabolic diseases. Polyphenols, naturally present in many foods (fruits, vegetables, cocoa, etc.) and beverages (tea, coffee, red wine, etc.), have been consistently reported as beneficial compounds, able to preserve metabolic homeostasis, mainly in animal models but also in several clinical studies (2–4). The low incidence of cardiovascular diseases in Mediterranean populations despite high consumption of lipids, commonly described as the “French paradox,” could be in part attributable to the consumption of polyphenol-rich diets, with olive oil, fruits, vegetables, and even red wine (5). In epidemiological studies, high intake of polyphenols was associated with lower risk of developing type 2 diabetes (6), and clinical trials in type 2 diabetic patients showed that purified anthocyanin could limit insulin resistance and increase anti-oxidative capacity (7). It was also reported that 14 days of coffee consumption preceding a 6 days fructose challenge was able to attenuate the HIR (8), and similarly, we have demonstrated that a dietary supplementation with grape polyphenol extracts for one month could prevent fructose induced oxidative stress and insulin resistance in high metabolic risk subjects (9). In heathy subjects, 15 days supplementation with dark chocolate increased insulin sensitivity (10), and 4-week supplementation with purified catechins decreased fasting insulin concentrations and HOMA-IR (11). A meta-analysis of randomized controlled trials (RCTs) performed in healthy or overweight subjects also highlighted a decrease in fasting insulin and HOMA-IR with cocoa supplementation, rich in polyphenols (12). However, several other studies did not show beneficial effect of polyphenols on metabolic parameters (13), indicating that additional studies investigating the effects of polyphenols in people submitted to a deleterious nutritional environment are still needed.

Experimental overfeeding simulates the early metabolic alterations induced by a caloric imbalance (14) and represents a useful model to precisely control food intake and to monitor weight changes, fat accumulation and metabolic parameters (15). For example, short term fat overfeeding was proven to induce insulin resistance and increased circulating CRP and MCP-1 in healthy volunteers (16). 56 days of high-fat overfeeding induced a remodeling of genes linked with fatty acid metabolism and mitochondria in skeletal muscle (17). Finally, another study showed that smaller fat cells at baseline was associated to worsened metabolic outcomes after overfeeding (17). Overfeeding can also be a good model to compare the effects of different diets. For example, overfeeding saturated or unsaturated fat has distinct effects on the liver and lipolysis (18). We therefore designed a one month randomized, double-blind, placebo-controlled trial to assess the ability of a daily nutritional amount of a grape polyphenol supplementation to counteract the consequences of a high calorie-high fructose overfeeding diet with 50% energy excess. The same study was conducted in parallel with men (in Lyon, France) and women (in Lausanne, Switzerland), with a specific focus on insulin sensitivity using a two-stage hyperinsulinemic-euglycemic clamp procedure and on post-prandial lipid metabolism using stable isotope labeled lipids in test meal.

## Research Design and Methods

### Study Design

**FIGURE 1:**
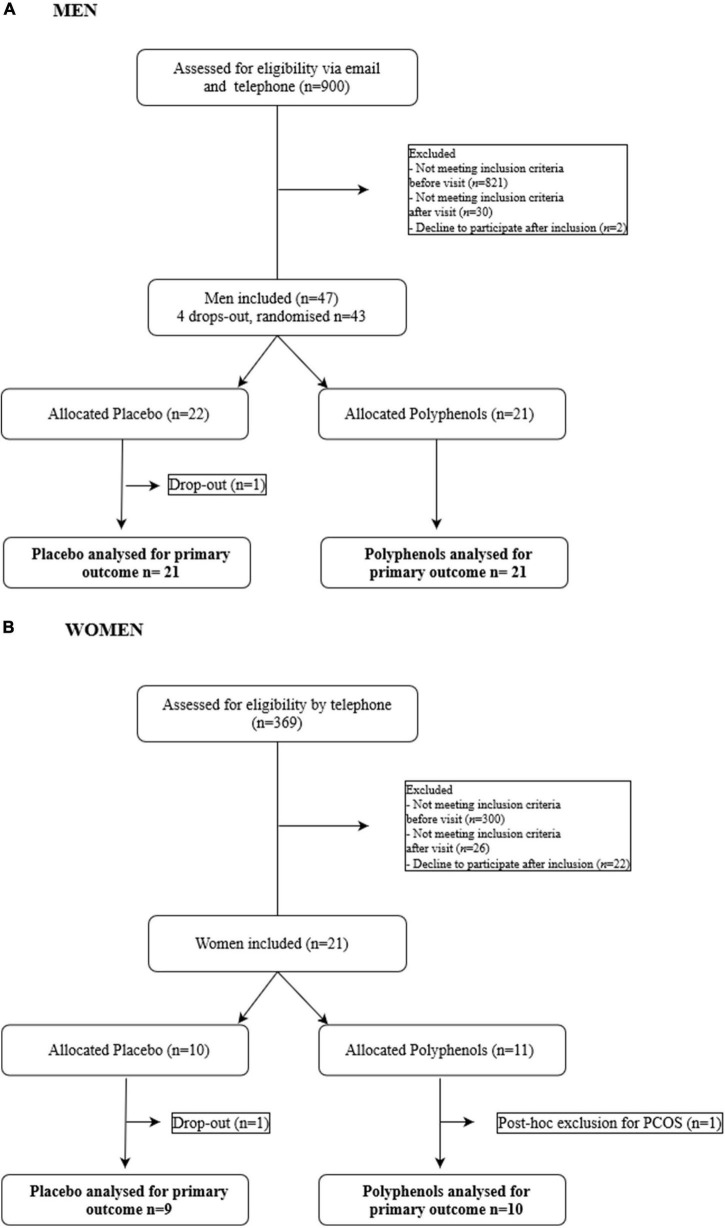
Flow chart of the study. **(A)** Men. **(B)** Women.

The full details of the clinical trial are described in the Supplementary Materials. The intervention study was an investigator-initiated randomized, double blind, placebo-controlled, parallel-group trial, with each participant serving as her or his own control. A timeline summarizing the study design is shown in Supplementary Figure 1. Participants were submitted to 31 days of overfeeding during which they were required to add to their usual daily diet, between their meals, a supplement of snack foods equating to a 50% excess of their total energy expenditure. The supplement was provided to the subjects in daily packages and was composed of a determined amount of soda, chocolate breads, chips and chocolate bars. In parallel, a supplementation of 2 g of grape polyphenols or placebo had to be taken each day by the subjects. Composition of the polyphenol extract is provided in the Supplementary Table 1. The study was conducted according to the CONSORT guidelines in Lyon, France (NCT02145780) for the male cohort and in Lausanne, Switzerland (NCT02225457) for the female cohort. The participant flow chart is shown in Figure 1. The protocol was approved in Lyon by the ethics committee of Lyon Sud-Est according to the French “Huriet-Serusclat” law, and in Lausanne by the regional committee for human experimentation (Vaud, Switzerland). All participants were given oral and written information before the protocol, and a written informed consent was obtained from all of them.

### Metabolic Explorations

Metabolic explorations were carried out in the morning, after an overnight fast on day 0 and on day 31, as described in Supplementary Figure 1. In women, clamps and test meals were performed during the follicular phase of the menstrual cycle.

Insulin sensitivity was assessed using a two-step hyperinsulinemic-euglycemic clamp (0.2 mUI.kg^–1^.min^–1^ and 1 mUI.kg^–1^.min^–1^) as previously described (19).

Metabolic fate of dietary lipids was investigated using a breakfast test meal enriched with 250mg [1,1,1-^13^C_3_]-triolein (99 atom% ^13^C, Eurisotop, Saint-Aubin, France). The isotopic enrichment of the lipid fractions was assessed as previously reported (20).

### Metabolites, Hormones and Polyphenols

Biochemicals were measured by routine methods as previously described (14).

Plasma polyphenol concentrations were analyzed at the metabolomic platform of IARC (International Agency for Research on Cancers, Lyon), using Ultra High Pressure Liquid Chromatography ElectrosprayIonization Tandem Mass Spectrometry (UHPLC-ESI–MS/MS) as described previously (21).

### Body Composition

Body composition was determined by whole-body dual-energy x-ray absorptiometry (in Lyon: Lunar Prodigy; in Lausanne: Lunar iDXA) before and after overfeeding.

### Adipose Tissue Distribution and Liver Fat Evaluation by MRI in Men

The visceral and subcutaneous fat volumes were quantified using three-dimensional MRI at 3T (Ingenia Philips, Andover, MA, United States) and the proton density fat fraction (PDFF) in the liver was quantified using MRS, as previously described (22) and used to assess the liver fat percentage.

### Physical Activity

Physical activity was monitored using Actigraph GT3X accelerometer (Actigraph, Pensacola, United States) before and during the overfeeding (D21-D28) and physical activity level (PAL) were determined.

### Adipocyte Size Measurement

Adipose tissue biopsies were processed as recommended for histological analysis (16). Pictures taken under a fluorescent microscope were analyzed with the ImageJ software to assess adipocyte diameter and area.

### Power Calculation

In men the sample size calculation was performed using PASS 2011 software (NCSS, LLC. Kaysville, Utah). The glucose infusion rate (GIR) obtained during similar euglycemic hyperinsulinemic clamp in a previous study (19) was used as reference for the calculation. Expecting a difference of at least 1 mg.kg^–1^.min^–1^ between the two groups, Placebo (PCB) and Polyphenol (PP), and with a 5% alpha error and a 80% power, 21 subjects per group were required. In women, the sample size calculation was based on a previous study (23) with a sample size of 9 subject per group.

### Statistical Analyses

Clinical, imaging and metabolic variables were reported as mean (± SEM), median (range) or number (%) as appropriate. The area under curves were calculated using the trapezoidal method. Between treatment group effect of overfeeding was analyzed with a repeated ANOVA analysis for Gaussian data or Mann Whitney tests for non-Gaussian data. The effect of the time and group in the male and female cohort were analyzed separately (Time, Time*group) with the same statistical method. If necessary, a logarithm transformation was considered depending on the distribution of variables. For non-parametric variables, diet effect was analyzed using Wilcoxon test. Homogeneity of variance was analyzed before performing parametric tests, with a Levene test. Correlations were assessed using Spearman rank correlation coefficients. *Post hoc* analysis was performed when appropriate. Statistical significance was considered for P values < 0.05 and all statistical analyses were performed using SAS 9.4 (SAS Institute Inc., Cary, NC, United States) and IBM SPSS Statistics for Windows (Version 20.0. Armonk, NY: IBM Corp) or R (version 1.4.17.17).

### Data and Resource Availability

All data generated or analyzed during this study are included in the published article (and its online Supplementary Materials). No applicable resources were generated or analyzed during the current study.

## Results

### Responses to Overfeeding and Effects of Polyphenols on Anthropometric and Metabolic Parameters

#### Male Study

Forty two volunteers, aged 31 ± 2 y, BMI: 24.9 ± 0.2 kg/m^2^ completed the study (Figure 1). The subjects in the placebo group and polyphenol group did not differ by age, BMI, body composition and biochemical characteristics (Table 1). During the overfeeding period, energy intake increased from 2252 ± 91 to 3348 ± 76 kcal/day (Supplementary Table 2). The spontaneous intake of dietary polyphenols was the same between the two groups before overfeeding, and did not change with overfeeding. Subjects maintained the same level of PAL (Before and after overfeeding 1.54% ± 0.12% and 1.55% ± 0.13% for the placebo group and 1.55% ± 0.18%, 1.54% ± 0.14% for the polyphenol group).

**TABLE 1:** Body composition, resting energy expenditure (REE), leptin, adiponectin, C-reactive protein (CRP), fasting glycemia, insulinemia, homeostasis model assessment of insulin resistance (HOMA-IR) and euglycemic hyperinsulinic clamp parameters of the male cohort and female cohort before and after overfeeding.

	Placebo		Polyphenol		p		Wilcoxon paired test	
				
	Baseline	Overfeeding	Baseline	Overfeeding	Time	Time*Group	Placebo	Polyphenol
Men (n =)	21		21					
Age	33 ± 2		29 ± 2					
Body Mass index (kg/m^2^)	25.1 ± 0.3	25.9 ± 0.3	24.8 ± 0.3	25.6 ± 0.3	‡	ns		
Body weight (kg)	79.8 ± 1.6	82.4 ± 1.6	80.1 ± 1.6	82.7 ± 1.7	‡	ns		
Fat mass (kg)	19.6 ± 1.2	21.2 ± 1.1	21.2 ± 1.2	23.0 ± 1.4	‡	ns		
Fat free mass (kg)	58.3 ± 1.1	58.9 ± 1.1	57.3 ± 1.3	57.8 ± 1.3	*	ns		
REE (kcal/day)	1715 ± 45	1771 ± 42	1773 ± 46	1820 ± 52	ns	ns		
Leptine (μg/L)	4.50 ± 0.61	6.92 ± 0.82	5.11 ±0.67	7.32 ± 0.92	‡	ns		
Adiponectine (pg/mL)	11.1 ± 1.3	13.3 ± 1.5	14.8 ± 1.8	18.6 ± 2.4	‡	ns		
CRP (mg/L)	3.49 ± 1.15	1.43 ± 0.36	2.60 ± 1.62	1.62 ± 0.48			ns	ns
Glycemia (mmol/L)	5.32 ± 0.06	5.30 ± 0.08	5.12 ± 0.07	5.14 ± 0.07	ns	ns		
Insulinemia (mUI/L)	7.54 ± 0.86	9.80 ± 0.77	6.75 ± 0.51	8.38 ± 0.85	‡	ns		
HOMA-IR	1.79 ± 0.20	2.38 ± 0.19	1.54 ± 0.12	1.94 ± 0.21	‡	ns		
Fasting EGP (mg/kg/min)	2.27 ± 0.07	2.16 ± 0.04	2.11 ± 0.05	2.16 ± 0.04			ns	ns
EGP inhibition by low insulin (%)	82.8 ± 2.6	86.4 ± 3.1	83.6 ± 3.4	85.9 ± 3.1	ns	ns		
Hepatic Insulin Resistance index	17.2 ± 2.0	21.3 ± 1.8	14.2 ± 1.1	18.4 ± 2.0	*	ns		
GIR (mg/kg/min)	8.26 ± 0.54	8.09 ± 0.61	8.84 ± 0.57	8.14 ± 0.49	ns	ns		
Women (n =)	9		10					
Age	24 ± 1		27 ± 1					
Body Mass index (kg/m^2^)	20.9 ± 0.5	22.0 ± 0.6	21.8 ± 0.6	22.8 ± 0.6	‡	ns		
Body weight (kg)	59.6 ± 2.2	62.7 ± 2.6	60.7 ± 2.9	63.5 ± 3.0	‡	ns		
Fat mass (kg)	16.5 ± 1.1	18.8 ± 1.2	19.7 ± 2.0	20.8 ± 2.0	‡	ns		
Fat free mass (kg)	41.6 ± 1.7	42.6 ± 1.7	39.4 ± 1.5	40.4 ± 1.4	*	ns		
REE (kcal/day)	1248 ± 45	1275 ± 34	1212 ± 34	1289 ± 30	ns	ns		
Leptine (μg/L)	9.08 ± 1.77	14.2 ± 1.8	9.13 ± 1.69	17.6 ± 3.2	†	ns		
Adiponectine (pg/mL)	15.97 ± 2.4	20.77 ± 2.3	16.31 ± 1.4	21.24 ± 2.0	†	ns		
CRP (mg/L)	0.50 ± 0.24	0.51 ± 0.11	0.72 ± 0.24	0.94 ± 0.26	ns	ns		
Glycemia (mmol/L)	5.09 ± 0.09	5.02 ± 0.10	4.94 ± 0.08	5.09 ± 0.09	ns	*		
Insulinemia (mUI/L)	6.33 ± 0.77	9.17 ± 1.04	6.35 ± 0.71	9.29 ± 1.43	‡	ns		
HOMA-IR	1.44 ± 0.19	2.08 ± 0.25	1.40 ± 0.16	2.05 ± 0.30	†	ns		
Fasting EGP (mg/kg/min)	2.46 ± 0.09	2.38 ± 0.06	2.20 ± 0.10	2.32 ± 0.08	ns	ns		
EGP inhibition by low insulin (%)	66.3 ± 3.9	63.9 ± 8.4	71.9 ± 8.6	68.0 ± 5.0	ns	ns		
Hepatic insulin Resistance index	15.6 ± 2.0	22.1 ± 2.8	13.6 ± 1.3	20.8 ± 2.6	†	ns		
GIR (mg/kg/min)	8.91 ± 0.95	9.63 ± 1.01	9.21 ± 0.72	9.27 ± 0.69	ns	ns		

EGP: Endogenous glucose production, GIR: Glucose infusion rate. *p < 0.05. †p < 0.01. ‡ p < 0.001. Data are expressed as mean ± SEM.

Polyphenols were analyzed in plasma showing a significant increase in several species and metabolites at D28 in the polyphenol group only, including resveratrol, catechin, homovanillic and gallic ethyl ester (Supplementary Table 1).

The nutritional intervention led to an increase in body weight (2.6 ± 0.3 kg and 2.5 ± 0.3 kg, pTime < 0.001) and fat mass (1.6 ± 0.3 kg and 1.7 ± 0.3 kg, pTime < 0.001) in placebo and polyphenol groups, with no difference between groups (Table 1). Fat mass gain represented 62 ± 1% of the total weight gain. Serum leptin and adiponectin increased similarly in the two groups in response to overfeeding, and C-reactive protein (CRP) was not affected in either group (Table 1). Additional metabolic parameters are presented in Supplementary Table 3. Overfeeding induced an increase in total cholesterol, HDL-cholesterol and LDL cholesterol (all with pTime < 0.05) in the two groups, without significant difference between placebo and polyphenol.

#### Female Study

Nineteen female subjects, aged 26 ± 1 y, BMI: 21.4 ± 0.4 kg/m^2^ completed the study. The subjects in the placebo and the polyphenol group were comparable regarding age, BMI, body composition and biochemical characteristics (Table 1). During the overfeeding period, energy intake increased on average from 1989 ± 63 to 3181 ± 97 kcal/day (Supplementary Table 2). There was no difference between the two groups in the spontaneous polyphenol intake. Subjects maintained the same level of PAL (Before and after overfeeding 1.59% ± 0.19% and 1.49% ± 0.09% for the placebo group and 1.69% ± 0.15% and 1.58% ± 0.14% for the polyphenol group).

The nutritional intervention led to an increase in body weight (3.1 ± 0.5 kg and 2.8 ± 0.4 kg, pTime < 0.001) and fat mass (1.8 ± 0.4 kg and 1.2 ± 0.2 kg, pTime < 0.001) in placebo and polyphenol groups, with no difference between groups (Table 1). Like for the men, serum leptin and adiponectin increased similarly in the two groups in response to overfeeding, and CRP was not affected (Table 1). Overfeeding induced an increase in total cholesterol and HDL-cholesterol (all with pTime < 0.05) in the two groups, without significant difference between placebo and polyphenol (Supplementary Table 3). There was a significant increase in alanine transaminase (ALAT) (pTime < 0.05) with no effect of polyphenols.

### Responses to Overfeeding and Effects of Polyphenols on Glucose Metabolism and Insulin Sensitivity

#### Male Study

Fasting glycemia did not change during the intervention, either upon overfeeding or during polyphenol consumption, while insulinemia increased (pTime < 0.001) in both the placebo and polyphenol groups (Table 1). Consequently, there was an increase in HOMA-IR (0.59 ± 0.18 for the placebo group and 0.39 ± 0.17 for the polyphenol group, pTime < 0.001) in the two groups, without significant difference between placebo and polyphenol. Overfeeding did not affect basal endogenous glucose production rate nor its inhibition at the first step of the clamp. However, overfeeding led to an increase in the HIR, both in placebo and polyphenol groups (4.09 ± 1.99 and 4.12 ± 1.70, respectively, pTime < 0.05), but without difference between groups. At the end of the second step of the clamp, GIR was unaffected by the overfeeding in both the placebo and the polyphenol groups.

#### Female Study

Overfeeding intervention did not affect fasting glycemia in the placebo group, but a significant, although moderate, increase (from 4.9 ± 0.1 to 5.1 ± 0.1 mmol/L, pTime*Group < 0.05) was observed in the polyphenol group (Table 1). Like for the men, the nutritional intervention led to an increase in fasting insulinemia in both groups (pTime < 0.001) and consequently, the HOMA-IR (+ 0.63 ± 0.13 for the placebo group and + 0.65 ± 0.22 for the polyphenol group, pTime < 0.01) increased, but without an effect of polyphenols. During the clamp, overfeeding did not affect endogenous glucose production rate, although there was an increase in HIR in both groups (6.47 ± 1.8 and 7.16 ± 2.1 for the placebo and polyphenol groups, respectively, pTime < 0.01), which was not ameliorated by polyphenol consumption (Table 1). Finally, as observed in men, GIR was not affected by overfeeding nor by polyphenol supplementation when measured at the second step of the hyperinsulinemic clamp.

### Responses to Overfeeding and Effects of Polyphenols on Postprandial Metabolism

#### Male Study

**FIGURE 2:**
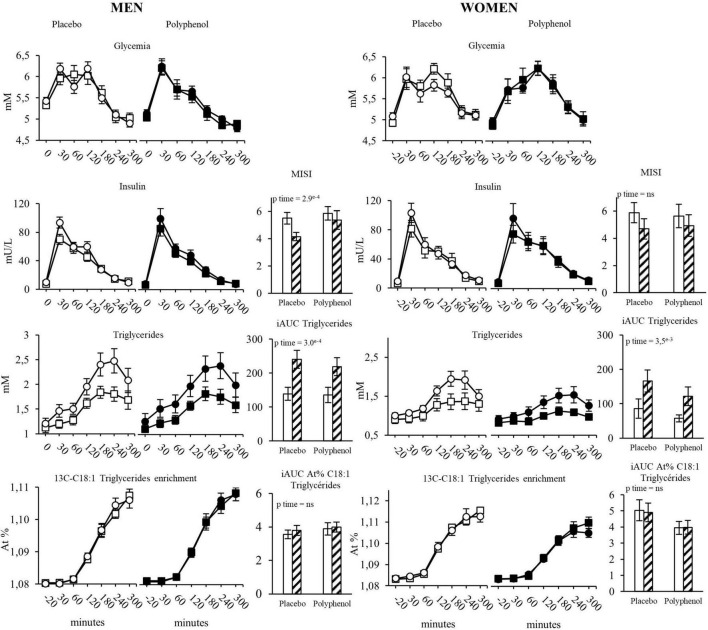
Evolution of glycemia, insulinemia, triglycerides and 13C-C18:1 triglycerides in plasma during the test meal, in men and women in the placebo and the polyphenol groups. MISI: Matsuda insulin sensitivity index, iAUC in each group are represented before and after overfeeding. White squares: Placebo group at baseline; White circles: Placebo group after overfeeding; Black squares: Polyphenol group at baseline; Black circles: Polyphenol group after overfeeding; White bars: Baseline; Hatched bars: Overfeeding. Data are expressed as mean ± SEM.

Postprandial metabolism was evaluated using a standardized test meal with labeled lipids (20). Regarding the evolution of glycemia and insulinemia during the postprandial phase, overfeeding led to an increase in the 5h incremental area under the curve (iAUC) of insulinemia with no effect on glycemia iAUC (Figure 2 and Supplementary Table 4), resulting accordingly in a significant decrease in Matsuda Insulin Sensitivity Index (MISI) in the two groups (pTime < 0.001), which was not corrected by polyphenol intake. Regarding the postprandial metabolism of lipids, overfeeding significantly increased the 5h iAUC of triglycerides (pTime < 0.001). Post-prandial ^13^C oleic acid enrichment (5h iAUC) remained unchanged with overfeeding in both groups. There was no impact on whole-body meal-derived fatty acids oxidation rate estimated from the isotopic enrichment of expired ^13^CO2 [iAUC Oxidized tracer (g): pTime = 0.6] (Supplementary Table 4).

#### Female Study

Overfeeding led to a decrease in the 5h iAUC of postprandial glycemia (pTime = 0.01) while the 5h iAUC of insulinemia remained stable, with no effect of polyphenols (Figure 2 and Supplementary Table 4). Overfeeding was associated with a significant decrease in the MISI without difference between groups (p Time = 0.03, p Time*Group = 0.6). Like in men, overfeeding led to an increase of the 5h iAUC of triglycerides (pTime < 0.001), with no impact of polyphenols (Figure 2). There was no change of post prandial plasma enrichment of ^13^C oleic acid and of whole-body meal-derived fatty acid oxidation (Supplementary Table 4), neither after overfeeding nor in the presence of polyphenols.

### Responses to Overfeeding and Effects of Polyphenols on Adipose Tissue Distribution and Adipocyte Size

**FIGURE 3:**
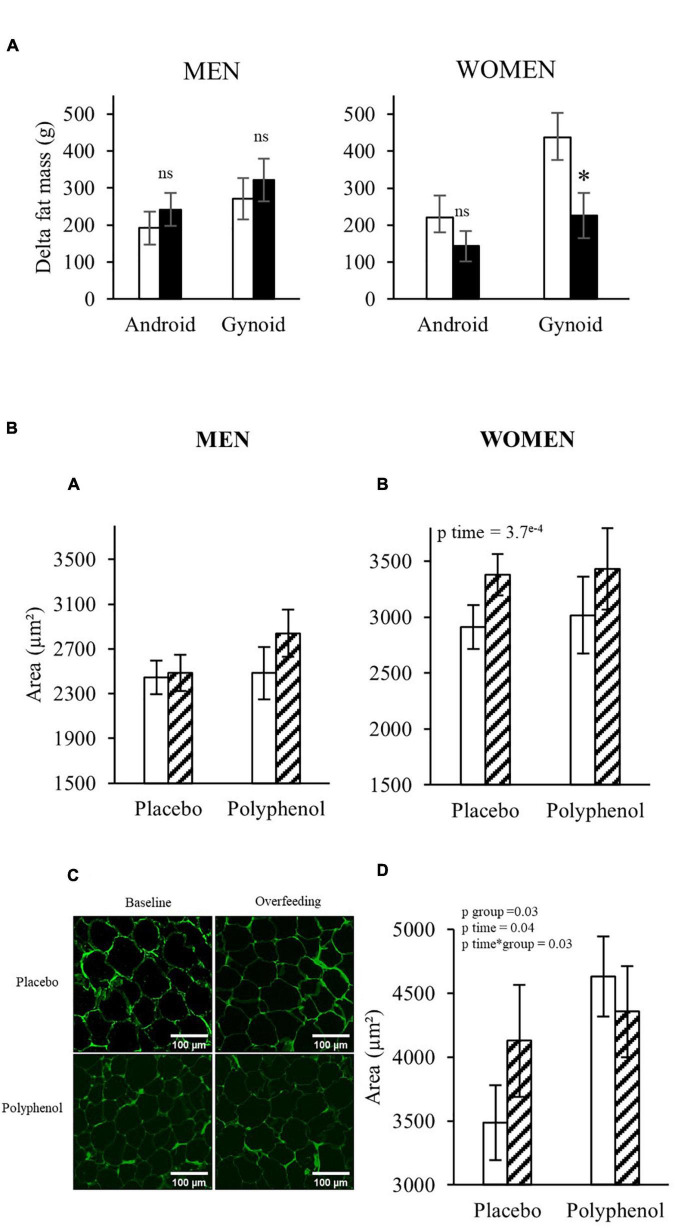
**(A)** Evolution of android and gynoid fat mass in both populations analyzed by DEXA. *T*-test or Wilcoxon, * *p* < 0.05. White bars: Placebo group; Black bars: Polyphenol group. Data are expressed as mean ± SEM. **(B)** Periumbilical adipocyte area in men **(A)** and women **(B)** and gluteal adipocyte area in women **(D)** before (D-7) and after overfeeding (D28) (μm^2^) in each group. Periombilical adipose tissue pictures of a subject in the placebo and polyphenol group at baseline and after overfeeding **(C)**. ANOVA with repeated measures or non- parametric test when appropriate were performed to study the effect of time and the effect of the polyphenol supplementation. No effect of Time*Group using ANOVA repeated measures, or non-parametric test, except for the Gluteofemoral adipocyte size in women. White bars: Baseline; Hatched bars: Overfeeding. Data are expressed as mean ± SEM.

Android and gynoid fat mass were assessed by DEXA in the two cohorts (Supplementary Table 5). Both adipose tissue depots significantly increased during overfeeding in both genders (pTime < 0.001). In men, polyphenol consumption did not impact the effect of overfeeding, whereas, in women, the supplementation reduced the overfeeding-induced gynoid fat mass (Figure 3A), which was less increased in the polyphenol group (226 ± 61 g) compared to the placebo (437 ± 70 g) (*p* < 0.05). No effects of polyphenol was observed on the android fat mass.

These DEXA data suggest that the polyphenol supplementation could eventually affect the accumulation of fat in some depots in women, therefore we checked adipose tissue cellularity by measuring the area of adipocytes in subcutaneous adipose tissue biopsies from abdominal and femoral regions. Figure 3B shows that while the adipocyte size in women increased during overfeeding in the periumbilical region in both groups (467 ± 115 μm^2^ and 349 ± 92 μm^2^ for the placebo and polyphenol group, respectively) and in the gluteofemoral depot for the placebo group (641 ± 367 μm^2^), it decreased in the gluteofemoral depot in the polyphenol group (−348 ± 198 μm^2^) (pTime*Group < 0.05). In men, neither overfeeding nor polyphenol supplementation modified the adipocyte size in the peri-umbilical region.

Responses to overfeeding and effects of polyphenols on MRI-assessed adipose tissue distribution and liver fat content in men.

When measured by MRI, both subcutaneous (+ 90.8 ± 22 cm^3^ and + 111.6 ± 23 cm^3^ for the placebo and polyphenol group, respectively), and abdominal fat volume (+ 128.5 ± 31 cm^3^ and + 88.7 ± 30 cm^3^ for the placebo and polyphenol groups, respectively) were significantly increased during overfeeding (Supplementary Table 5), without difference between both groups. Using MR spectroscopy, we were able to assess liver fat, which was significantly increased in the placebo and the polyphenol groups, without effect of the polyphenol supplementation (+ 1.74% ± 0.5% and + 1.35% ± 0.3% for the placebo and polyphenol groups, respectively, pTime < 0.001). Of note, using Spearman correlations in the whole male cohort (*n* = 42), we found associations between the increase in liver fat content and the increase in ALAT (*R* = 0.42, *p* = 0.008) and a tendency with the increased HIR (*R* = 0.31, *p* = 0.06) (data not shown).

## Discussion

The present study is the first evaluation of the effect of a polyphenol extract supplementation on an experimental overfeeding in humans. Our objective was to test a potential protective role of a grape polyphenol mixture (2 g/day) on the metabolic and anthropometric adaptations to one month of overfeeding in healthy men and women participating in a similar randomized, placebo-controlled, parallel-group clinical trial.

Overfeeding experiments have been used previously to characterize the early changes occurring during weight gain, allowing the description of the kinetics of adipose tissue expansion (14), the modification of fat inflammation (16) or the adaptation of energy metabolism in skeletal muscle (24). Several studies investigated ultraprocessed food (25, 26), however, most of the overfeeding interventions have been performed with high fat diets, that do not really reflect actual food habits, and it was shown in several (16–18, 27) but not all (28, 29) studies that high-fat overfeeding can lead to reduced insulin sensitivity. For the present study, we designed a high calorie-high fructose overfeeding protocol, with a daily energy excess of + 50% of the total energy expenditure, provided by ultra-processed food such as soda, chocolate breads, chips and chocolate bars to mimic the western diet (30). Overfeeding for one month increased body weight and fat mass similarly in men and in women, and, measured by MRI in men only, increased visceral and subcutaneous fat depots and lipid deposition in the liver. The primary outcome of the trials was the effect of polyphenol supplementation on whole-body insulin sensitivity. In both genders, overfeeding promoted a slight deterioration of insulin sensitivity estimated by the HOMA-IR and the MISI, mainly related to an increase in fasting plasma insulin concentrations. The HIR was also increased after overfeeding and was slightly associated, at least in men, to liver fat accumulation estimated by MRI. However, polyphenol intake did not modify significantly any of these values. In addition, when assessed by euglycemic hyperinsulinemic clamp, overfeeding was not associated with alteration of insulin actions measured at both steps of the clamp (inhibition of hepatic glucose production and stimulation of whole-body glucose uptake). In this situation, polyphenol did not modify insulin responses estimated by the clamp, either in men, or in women.

Other studies have reported effects of polyphenol supplementation on insulin sensitivity markers, with a decreased HOMA-IR in obese adults fed with polyphenol enriched food (31) or supplemented with resveratrol (32). In heathy subjects, consumption of polyphenol rich food (chocolate) or purified compounds (catechins) has been also associated with improved insulin sensitivity (10–12). In a previous study, we found that first-degree relatives of type 2 diabetes patients were protected against fructose-induced insulin resistance when supplemented with the same grape polyphenol extract as the one used here (9). However, the protocol was different with polyphenol supplementation provided for two months before a 6 day challenge test with fructose. The design of the present trials, with consumption of polyphenol supplements during the overfeeding intervention, may have potentially concealed the effects that could have taken place when used as a preventive strategy, or when provided without drastically changing the diet (12, 31, 32). It should also be mentioned that the metabolic alterations in response to the present overfeeding experiment were not very large and thus could not be markedly improved by the polyphenol supplementation. However, in line with our observation, several other trials have also found that polyphenols did not modify insulin sensitivity and metabolic parameters (33), including studies with resveratrol (13), the prototypic polyphenol molecule for which a large bulk of pre-clinical data support positive actions on insulin sensitivity (34).

Overfeeding with a high calorie-high fructose diet was associated with an increase in liver fat content assessed by MRS analysis in men. Unfortunately, we did not have access to the liver fat content in women, but ALAT, a surrogate marker of liver fat (35), increased in women, suggesting that the evolution of liver fat was probably similar in both genders during overfeeding. Liver fat deposition could be the consequence of increased dietary fat intake and/or stimulation of hepatic *de novo* lipogenesis (36). A recent study agrees with this hypothesis as 7 weeks of overfeeding of sweetened beverages showed that fructose and sucrose but not glucose increased *de novo* lipogenesis (37). The high fructose content of the overfeeding diet, that led to an increase in circulating fructose, may have promoted *de novo* lipogenesis (5). In parallel, post-prandial triglycerides were elevated after overfeeding suggesting increased intestinal uptake of lipids. However, the analysis of the fate of dietary lipids during the test meals revealed a similar enrichment of labeled fatty acids in plasma before and after overfeeding. This indicates that the increase in post-prandial triglycerides levels was the result of an increase in both endogenous triglycerides from *de novo* lipogenesis and intestinal absorption of dietary lipids. This could be due to a concomitant increase of both processes or more likely to a decrease in triglycerides clearance. The composition of the supplementary diet, with a high content of saturated fats, could have contributed to increased intrahepatic triglyceride content (9, 10) and potentially also to hepatic insulin resistance (18). Several high saturated fat overfeeding studies agree with this statement. Feeding muffins high in saturated fatty acids for 7 weeks increased liver fat measured by MRI (38). Another overfeeding of saturated, unsaturated or simple sugars showed that 3 weeks of saturated fat is enough to increase intrahepatic triglycerides (18). Another study showed that overfeeding healthy men for 7 days with a high-fat high-fructose diet increased to a greater extent hepatic lipid content, compared to a high-fat or a high-fructose diet alone (39). Therefore, the increase in liver fat of our volunteers might be caused by a synergistic action of both fat and fructose.

In the female group, we observed a slight increase in fasting glycemia with the polyphenol supplementation compared to the placebo group. A meta-analysis of nine clinical trials showed that resveratrol consumption could be associated with increased fasting glucose in patients with type II diabetes mellitus (40). This effect on fasting glycemia in women further showed that the polyphenol supplementation had no beneficial effect in the trial, and could even be associated with a deleterious effect in the context of an hypercaloric diet, as already suggested (41).

Concerning body composition, overfeeding led to an increase in body weight and fat mass, and polyphenol supplementation did not protect against weight and fat mass gain in the condition of positive energy balance neither in men nor in women. However, although gynoid fat mass accumulation was observed in both genders, the gain was significantly lower in the polyphenol group compared to the placebo in the women. This difference in gynoid fat mass gain in women was associated with a decrease in adipocyte size in the subcutaneous gluteo-femoral adipose tissue. No effect was found in the abdominal subcutaneous depots in women or men. A recent study reported a reduction in adipocyte size in subcutaneous adipose tissue of obese individuals after 30 days of resveratrol supplementation (42), but the same group showed no effect using a combination of epigallocatechin-gallate and resveratrol for 3 months (32). In *in vitro* and in pre-clinical models, polyphenols demonstrated anti-lipogenic and pro-lipolytic action in adipocytes (43–46) leading to adipocyte size reduction (47). Our data suggest that polyphenol supplementation may modulate the accretion of gynoid fat in overfed women potentially through an impact on adipocyte size.

A strength of the study is the evaluation of the effects of overfeeding and of polyphenols both in men and in women. However, one limitation is the fact that despite being very similar, the two trials were performed in different countries with some differences in the protocols, that did not allow to combine the subjects to test directly for gender effects. Nevertheless, the results clearly showed that for most of the measured variables, both men and women responded very similarly to the intervention and the polyphenol supplementation.

In conclusion, the presented RCTs demonstrated that the supplementation with a grape polyphenol extract during one month of high calorie-high fructose overfeeding mimicking the western diet, had no major effect on insulin sensitivity, anthropometric or metabolic parameters. Overfeeding led to mild hepatic insulin resistance and increased postprandial triglycerides and liver fat, similarly in men and women. These parameters were not affected by polyphenol supplementation. Nonetheless, in women, polyphenol appeared to limit the accretion of fat mass and the size of adipocytes in the gynoid adipose tissue depot.

## Data Availability Statement

Data of this study are protected under the protection of health data regulation set by the French National Commission on Informatics and Liberty (Commission Nationale de l’Informatique et des Libertés, CNIL). According to French law on the publication of biomedical research/clinical trials, we are not allowed to make the clinical database publicly available on the web, nor to make visible the location of the study associated with the database, nor to send the clinical database to third parties. The anonymized data that support the findings of the present study can be available upon reasonable request to the corresponding author.

## Ethics Statement

The studies involving human participants were reviewed and approved by The Comité de Protection des Personnes SUD-EST IV 13/058 and Commission cantonale d’éthique de la recherche sur l’être humain du canton de Vaud (CER-VD 391/13). The patients/participants provided their written informed consent to participate in this study.

## Author Contributions

ML, NV, FP, and HV designed the study. AN, NV, PD, KS, ED, J-AN, SL-P, LM, VS, KB-P, HR, OB, N-HY, KB, AB, BS, and MG performed the study and analyzed the data. BS, PD, HV, EM, and ML analyzed and prepared the data for publication and wrote the manuscript. ML was the guarantor of this work and, as such, had full access to all the data in the study and takes responsibility for the integrity of the data and the accuracy of the data analysis. All authors read and approved the submitted version of the manuscript.

## Conflict of Interest

The authors declare that the research was conducted in the absence of any commercial or financial relationships that could be construed as a potential conflict of interest.

## Publisher’s Note

All claims expressed in this article are solely those of the authors and do not necessarily represent those of their affiliated organizations, or those of the publisher, the editors and the reviewers. Any product that may be evaluated in this article, or claim that may be made by its manufacturer, is not guaranteed or endorsed by the publisher.

## Appendix

Participating investigators in France:

- Martine Laville
- Emmanuel Disse
- Nathalie Feugier-Favier
- Stéphanie Lambert-Porcheron

Participating investigators in Switzerland:

- Nathalie Vionnet
- François Pralong
- Naba-al-Huda Yassin
- Weilin Kong
- Magali Van Lecwick
- Ismael Turk
