# Supplementary material for: Polyphenol Supplementation Did Not Affect Insulin Sensitivity and Fat Deposition During One-Month Overfeeding in Randomized Placebo-Controlled Trials in Men and in Women
Source: Front Nutr. 2022 May 9;9:854255. doi: 10.3389/fnut.2022.854255 (PMC9125251; doi:10.3389/fnut.2022.854255)
Supplement: Supplementary file 1 [file Data_Sheet_1.docx]

1. **Supplementary figures**


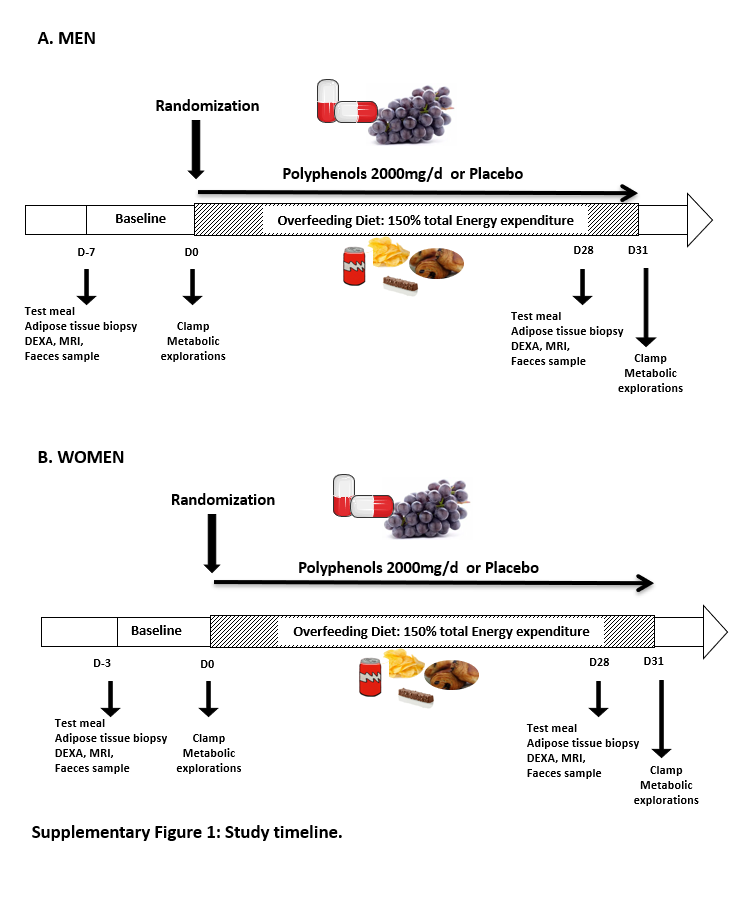


1. **Supplementary tables**

| **Composition of polyphenols supplements** | **%** | **mg/day** |
| --- | --- | --- |
| Total polyphenols equivalent catechin method DO 280 nm | 101 | 2020 |
| Procyanidins equivalent catechinVanillin method | 18 | 360 |
| Anthocyans Disulfite decoloration method | 2 | 40 |
| Resveratrol equivalent trans-resveratrol HPLC method | 0.04 | 0.9 |

|  | **Placebo** | | | | | | **Polyphenol** | | | | | | **p** | | | **Wilcoxon Paired test** | |
| --- | --- | --- | --- | --- | --- | --- | --- | --- | --- | --- | --- | --- | --- | --- | --- | --- | --- |
|  | **Baseline** | | | **Overfeeding** | | | **Baseline** | | | **Overfeeding** | | | **Group** | **Time** | **Time*Group** | **Placebo** | **Polyphenol** |
| 3.4-di-OH-phenylpropionic (nM) | 30.4 | ± | 5.0 | 36.4 | ± | 14.2 | 20.1 | ± | 2.1 | 26.0 | ± | 2.6 | † | * | ‡ |  |  |
| Epigallocatechin (nM) | 15.2 | ± | 3.2 | 32.5 | ± | 5.8 | 8.86 | ± | 0.35 | 7.26 | ± | 0.78 | ns | ‡ | ‡ |  |  |
| 3-OH-phenylacetic (nM) | 54.3 | ± | 9.8 | 47.7 | ± | 9.7 | 45.1 | ± | 14.3 | 151.4 | ± | 27 | ns | ns | ‡ |  |  |
| Resveratrol (nM) | 1.95 | ± | 0.07 | 2.99 | ± | 0.86 | 3.29 | ± | 0.64 | 12.0 | ± | 2.4 | ns | ns | ‡ |  |  |
| Isorhamnetin (nM) | 29.9 | ± | 2.0 | 28.4 | ± | 1.4 | 25.5 | ± | 1.6 | 35.1 | ± | 3.3 | ns | ns | ‡ |  |  |
| 3.5-di-OHphenylpropionic (nM) | 37.9 | ± | 4.5 | 28.8 | ± | 3.3 | 26.7 | ± | 2.9 | 33.2 | ± | 5.3 | * | † | † |  |  |
| 3.4-di-OH-phenylacetic (nM) | 21.1 | ± | 1.7 | 22.5 | ± | 1.4 | 19.8 | ± | 3.3 | 26.5 | ± | 3.1 | ns | ns | † |  |  |
| Enterolactone (nM) | 9.34 | ± | 1.50 | 7.84 | ± | 2.0 | 11.3 | ± | 3.3 | 12.6 | ± | 2.6 | ns | * | † |  |  |
| Homovanillic (nM) | 55.4 | ± | 5.3 | 54.2 | ± | 3.2 | 53.0 | ± | 3.2 | 69.6 | ± | 6.5 | ns | ns | * |  |  |
| Gallic Ethyl Ester (nM) | 0.88 | ± | 0.11 | 0.82 | ± | 0.09 | 0.98 | ± | 0.19 | 11.7 | ± | 2.8 | ns | ns | * |  |  |
| Protocatechuic (nM) | 211 | ± | 7 | 204 | ± | 7 | 199 | ± | 6 | 207 | ± | 12 | ns | ns | * |  |  |
| Catechin (nM) | 19.5 | ± | 3.9 | 7.76 |  |  | 9.12 | ± | 0.61 | 36.2 | ± | 7.9 | ns | ns | * |  |  |
| Tyrosol (nM) | 2.08 | ± | 0.33 | 2.45 | ± | 0.24 | 2.49 | ± | 0.47 | 2.22 | ± | 0.37 | ns | * | ns |  |  |
| 3-OH-benzoic (nM) | 19.1 | ± | 10.3 | 14.8 | ± | 5.2 | 7.88 | ± | 1.47 | 23.9 | ± | 6.0 |  |  |  | ns | ‡ |
| Gallic (nM) | 17.8 | ± | 3.9 | 15.0 | ± | 1.6 | 12.6 | ± | 0.2 | 15.0 | ± | 1.1 |  |  |  | ns | * |

**Supplementary Table 1: Polyphenolic composition of the red grape extract used during the study and plasma polyphenol metabolites before and after intervention in the placebo group and the polyphenol group in men.** * p<0.05. † p<0.01. ‡ p<0.001. Data are expressed as mean ±SEM.

|  | **Placebo** | | | | | | **Polyphenol** | | | | | | **p** | | | **p** | |
| --- | --- | --- | --- | --- | --- | --- | --- | --- | --- | --- | --- | --- | --- | --- | --- | --- | --- |
|  | **Baseline** | | | **Overfeeding** | | | **Baseline** | | | **Overfeeding** | | | **Group** | **Time** | **Time*group** | **Placebo** | **Polyphenol** |
| **Men (n=)** | 21 | | |  | | | 21 | | |  | | |  |  |  |  |  |
| Energy intake (kcal/day) | 2227 | ± | 147 | 3322 | ± | 103 | 2277 | ± | 111 | 3374 | ± | 115 | ns | ‡ | ns |  |  |
| Proteins (%) | 16.7 | ± | 0.4 | 12.3 | ± | 0.3 | 15.5 | ± | 0.5 | 11.0 | ± | 0.3 | * | ‡ | ns |  |  |
| Proteins (g) | 92.0 | ± | 5.7 | 102 | ± | 4 | 88.0 | ± | 4.3 | 92.7 | ± | 4.1 | ns | * | ns |  |  |
| Lipids (%) | 35.8 | ± | 0.8 | 35.1 | ± | 0.7 | 36.0 | ± | 0.8 | 35.8 | ± | 0.6 | ns | ns | ns |  |  |
| SFA-MUFA-PUFA (%) | 36-35-12 | | | 38-42-11 | | | 37-33-12 | | | 40-41-10 | | | ns | ns | ns |  |  |
| Lipids (g) | 88.2 | ± | 6.2 | 130 | ± | 5 | 91.0 | ± | 5.0 | 135 | ± | 6 | ns | ‡ | ns |  |  |
| Carbohydrate (%) | 44.1 | ± | 1.1 | 50.4 | ± | 0.8 | 43.8 | ± | 1.0 | 50.9 | ± | 0.9 | ns | ‡ | ns |  |  |
| Carbohydrate (g) | 248 | ± | 18 | 419 | ± | 15 | 249 | ± | 12 | 427 | ± | 14 | ns | ‡ | ns |  |  |
| Fructose (g/day) | 14.7 | ± | 2.1 | 63.1 | ± | 1.6 | 11.2 | ± | 1.4 | 65.2 | ± | 1.6 | ns |  |  | ‡ | ‡ |
| Spontaneous polyphenol intake (mg) | 453 | ± | 59 | 521 | ± | 91 | 479 | ± | 89 | 427 | ± | 79 | ns | ns | ns |  |  |
| **Women (n=)** | 9 | | |  | | | 10 | | |  | | |  |  |  |  |  |
| Energy intake (kcal/day) | 2075 | ± | 89 | 3429 | ± | 128 | 1903 | ± | 84 | 2933 | ± | 90 | ns | ‡ | ns |  |  |
| Proteins (%) | 14.7 | ± | 1.6 | 11.0 | ± | 0.7 | 13.6 | ± | 1.3 | 12.3 | ± | 0.8 | ns | † | ns |  |  |
| Proteins(g) | 78.1 | ± | 11 | 95.4 | ± | 7.7 | 64.9 | ± | 6.6 | 89.5 | ± | 5.7 | ns | ns | ns |  |  |
| Lipids (%) | 33.9 | ± | 1.4 | 33.3 | ± | 1.1 | 33.8 | ± | 1.7 | 33.2 | ± | 0.8 | ns | ns | ns |  |  |
| SFA-MUFA-PUFA (%) | 38-31-12 | | | 37-36-10 | | | 40-30-11 | | | 38-34-10 | | | ns | ns | ns |  |  |
| Lipids(g) | 78.8 | ± | 5.9 | 127 | ± | 6 | 71.0 | ± | 3.6 | 109 | ± | 5 | ns | ‡ | ns |  |  |
| Carbohydrate (%) | 46.8 | ± | 2.7 | 51.9 | ± | 1.4 | 49.5 | ± | 2.6 | 51.7 | ± | 0.8 | ns | * | ns |  |  |
| Carbohydrate(g) | 240 | ± | 10 | 444 | ± | 17 | 237 | ± | 19 | 379 | ± | 14 | ns | ‡ | * |  |  |
| Fructose (g/day) | 14.0 | ± | 2.7 | 63.8 | ± | 3.1 | 19.4 | ± | 3.9 | 54.0 | ± | 2.3 | ns | ‡ | † |  |  |
| Spontaneous polyphenol intake (mg) | 415 | ± | 95 | 407 | ± | 97 | 534 | ± | 129 | 363 | ± | 92 | ns | ns | ns |  |  |

**Supplementary Table 2: Composition of the diet before and during the overfeeding for men and women.** * p<0.05. † p<0.01. ‡ p<0.001. Data are expressed as mean ±SEM.

|  | **Placebo** | | | | | | **Polyphenol** | | | | | | **p** | |
| --- | --- | --- | --- | --- | --- | --- | --- | --- | --- | --- | --- | --- | --- | --- |
|  | **Baseline** | | | **Overfeeding** | | | **Baseline** | | | **Overfeeding** | | | **Time** | **Time*Group** |
| **Men (n=)** | 21 | | |  | | | 21 | | |  | | |  |  |
| Triglycerides(mM) | 1.12 | ± | 0.10 | 1.07 | ± | 0.09 | 1.11 | ± | 0.08 | 1.11 | ± | 0.08 | ns | ns |
| NEFA(µM) | 384 | ± | 31 | 322 | ± | 19 | 453 | ± | 38 | 380 | ± | 32 | ns | ns |
| Total cholesterol(mM) | 4.43 | ± | 0.15 | 4.75 | ± | 0.18 | 4.37 | ± | 0.20 | 4.52 | ± | 0.24 | * | ns |
| HDL cholesterol(mM) | 1.08 | ± | 0.03 | 1.18 | ± | 0.05 | 1.11 | ± | 0.04 | 1.19 | ± | 0.04 | ** | ns |
| *LDL cholesterol*(mM) | 2.87 | ± | 0.13 | 3.09 | ± | 0.15 | 2.75 | ± | 0.17 | 2.83 | ± | 0.20 | * | ns |
| ALAT(UI/L) | 30.7 | ± | 2.1 | 45.8 | ± | 7.2 | 30.4 | ± | 3.2 | 50.7 | ± | 12.0 | ns | ns |
| TNF(pg/mL) | 7.22 | ± | 0.56 | 7.36 | ± | 0.65 | 6.41 | ± | 0.50 | 6.11 | ± | 0.42 | ns | ns |
| Uric Acid(µM) | 328 | ± | 9 | 342 | ± | 12 | 345 | ± | 13 | 371 | ± | 18 | ns | ns |
| **Women (n=)** | 9 | | |  | | | 10 | | |  | | |  |  |
| Triglycerides(mM) | 0.77 | ± | 0.05 | 0.86 | ± | 0.14 | 0.66 | ± | 0.06 | 0.71 | ± | 0.07 | ns | ns |
| NEFA(µM) | 420 | ± | 47 | 312 | ± | 50 | 526 | ± | 46 | 332 | ± | 41 | ns | ns |
| Total cholesterol(mM) | 3.69 | ± | 0.20 | 4.07 | ± | 0.31 | 3.57 | ± | 0.21 | 3.75 | ± | 0.17 | * | ns |
| HDL cholesterol (mM) | 1.38 | ± | 0.08 | 1.50 | ± | 0.08 | 1.27 | ± | 0.09 | 1.40 | ± | 0.12 | * | ns |
| LDL cholesterol (mM)) | 1.95 | ± | 0.22 | 2.17 | ± | 0.24 | 1.99 | ± | 0.15 | 2.04 | ± | 0.12 | ns | ns |
| ALAT(UI/L) | 18.8 | ± | 2.1 | 36 | ± | 9.9 | 18.4 | ± | 1.7 | 25.0 | ± | 2.1 | * | ns |
| TNF(pg/mL) | 9.10 | ± | 3.49 | 6.93 | ± | 1.23 | 5.46 | ± | 0.83 | 6.03 | ± | 1.12 | ns | ns |
| Estradiol(nmol/L) | 0.25 | ± | 0.05 | 0.27 | ± | 0.04 | 0.21 | ± | 0.02 | 0.29 | ± | 0.05 | ns | ns |

**Supplementary Table 3:** **Fasting metabolic parameters of the male and female cohort before and after overfeeding.** * p<0.05. † p<0.01. ‡ p<0.001. Data are expressed as mean ±SEM.

|  | **Placebo** | | | | | | **Polyphenol** | | | | | | **p** | | |
| --- | --- | --- | --- | --- | --- | --- | --- | --- | --- | --- | --- | --- | --- | --- | --- |
|  | **Baseline** | | | **Overfeeding** | | | **Baseline** | | | **Overfeeding** | | | **Group** | **Time** | **Time*group** |
| **Men (n=)** | 21 | | |  | | | 21 | | |  | | |  |  |  |
| iAUC Glycemia (mM.min) | 85 | ± | 36 | 52 | ± | 31 | 85 | ± | 23 | 76 | ± | 25 | ns | ns | ns |
| iAUC Insulinemia(mU.min/L) | 8258 | ± | 914 | 9094 | ± | 947 | 7737 | ± | 827 | 8897 | ± | 1088 | ns | ns | ns |
| iAUC NEFA (µM.min) | -42297 § | ± | 9120 | -34755 | ± | 7010 | -74766 § | ± | 10946 | -35781 | ± | 6680 | ‡ | ns | * |
| iAUC Triglycerides (mM.min) | 139 | ± | 19 | 241 | ± | 27 | 136 | ± | 22 | 219 | ± | 27 | ns | ‡ | ns |
| iAUC C18:1 Enrichment (%) | 3.6 | ± | 0.26 | 3.8 | ± | 0.30 | 3.9 | ± | 0.38 | 4.0 | ± | 0.30 | ns | ns | ns |
| iAUC Oxidized tracer (g) | 28.4 | ± | 2.7 | 29.5 | ± | 2.1 | 33.3 | ± | 3.4 | 30.6 | ± | 3.1 | ns | ns | ns |
| **Women** | 9 | | |  | | | 10 | | |  | | |  |  |  |
| iAUC Glycemia (mM.min) | 228 | ± | 37 | 137 | ± | 17 | 239 | ± | 31 | 204 | ± | 23 | ns | † | ns |
| iAUC Insulinemia(mU.min/L) | 9310 | ± | 1541 | 9356 | ± | 1234 | 10164 | ± | 1643 | 10562 | ± | 1633 | ns | ns | ns |
| iAUC NEFA (µM.min) | -105183 | ± | 21980 | -63223 | ± | 13512 | -92205 | ± | 9029 | -71292 | ± | 10442 | ns | * | ns |
| iAUC Triglycerides (mM.min) | 95 | ± | 30 | 198 | ± | 28 | 52 | ± | 10 | 119 | ± | 24 | ns | ‡ | ns |
| iAUC C18:1 Enrichment (%) | 5.0 | ± | 0.65 | 4.9 | ± | 0.58 | 3.9 | ± | 0.40 | 4.0 | ± | 0.42 | ns | ns | ns |
| iAUC Oxidized tracer (g) | 30.2 | ± | 2.8 | 25.2 | ± | 2.2 | 23.9 | ± | 2.8 | 21 | ± | 3 | ns | ns | ns |

**Supplementary Table 4: Test meal: evolution of 5h iAUC of glycemia. insulinemia. NEFA. triglycerides. C18:1 Triglycerides enrichment and of 6h iAUc Oxidized tracer in breath during the test meal in men and women.** * p<0.05. † p<0.01. ‡ p<0.001. § p<0.05 between groups at baseline. Data are expressed as mean ±SEM.

|  | **Placebo** | | | | | | **Polyphenol** | | | | | | **p** | |
| --- | --- | --- | --- | --- | --- | --- | --- | --- | --- | --- | --- | --- | --- | --- |
|  | **Baseline** | | | **Overfeeding** | | | **Baseline** | | | **Overfeeding** | | | **Time** | **Time*Group** |
| **Men (n=)** | 21 | | |  | | | 21 | | |  | | |  |  |
| Waist circumference (cm) | 91.0 | ± | 1.2 | 94.0 | ± | 1.3 | 91.9 | ± | 1.2 | 94.2 | ± | 1.5 | ‡ | ns |
| Android Fat mass (kg) | 1.89 | ± | 0.15 | 2.09 | ± | 0.14 | 2.07 | ± | 0.13 | 2.31 | ± | 0.15 | ‡ | ns |
| Gynoid fat mass (kg) | 3.65 | ± | 0.20 | 3.92 | ± | 0.20 | 3.93 | ± | 0.24 | 4.26 | ± | 0.27 | ‡ | ns |
| MRI Abdominal subcutaneous AT(cm^3^) | 1104 | ± | 74 | 1191 | ± | 81 | 1173 | ± | 86 | 1293 | ± | 107 | ‡ | ns |
| MRI Visceral AT volume (cm^3^) | 922 | ± | 104 | 1070 | ± | 116 | 924 | ± | 83 | 997 | ± | 104 | ‡ | ns |
| Liver fat (%) | 1.20 | ± | 0.23 | 3.02 | ± | 0.61 | 2.81 | ± | 1.26 | 3.91 | ± | 1.53 | ‡ | ns |
| **Women (n=)** | 9 | | |  | | | 10 | | |  | | |  |  |
| Waist circumference (cm) | 77.3 | ± | 1.7 | 82 | ± | 2.1 | 78.2 | ± | 2.2 | 82.0 | ± | 2.7 | ‡ | ns |
| Android Fat mass (kg) | 0.87 | ± | 0.11 | 1.12 | ± | 0.14 | 1.11 | ± | 0.16 | 1.26 | ± | 0.15 | ‡ | ns |
| Gynoid fat mass (kg) | 3.41 | ± | 0.26 | 3.96 | ± | 0.33 | 4.05 | ± | 0.34 | 4.27 | ± | 0.33 | ‡ | * |

**Supplementary Table 5: Body composition characteristics of the male and female cohort before and after overfeeding. Abdominal MRI of the male cohort before and after overfeeding.** * p<0.05. † p<0.01. ‡ p<0.001. Data are expressed as mean ±SEM.
